# Twenty‐Year Progress in Landscape Genetics of Tetrapods

**DOI:** 10.1002/ece3.74145

**Published:** 2026-08-05

**Authors:** Tongtong Gu, Xing Jia, Yuxin He, Zixin Lin

**Affiliations:** ^1^ School of Life Sciences Yunnan Normal University Kunming China; ^2^ State Key Laboratory for Conservation and Utilization of Bio‐Resource in Yunnan, School of Life Sciences Yunnan University Kunming China

**Keywords:** conservation, functional connectivity, genomics, landscape genetics, population structure, tetrapods

## Abstract

Landscape genetics, an interdisciplinary field integrating population genetics, landscape ecology, and spatial analytical techniques, has progressed significantly over two decades, yet systematic reviews of its application to tetrapods remain limited. Here, we review 385 studies published between 2003 and 2025, covering 434 species across eight geographic regions, to assess taxonomic and geographic bias, common research questions, analytical approaches, and key environmental drivers of genetic variation. Our results show that Anura and Passeriformes are the most frequently studied groups, while threatened species were particularly poorly represented. North America dominates geographically, followed by Europe, while Asia, South America, Africa, and Oceania remain poorly sampled. Most studies investigate genetic connectivity and population structure, frequently employing Mantel tests, AMOVA, and STRUCTURE, although recent work increasingly applies SNP‐based genomic tools to detect both neutral and adaptive variation. Habitat permeability, topography, and artificial landscapes consistently influence genetic variation. These findings emphasize the need for expanded research across diverse tetrapod taxa and ecosystems, with particular attention to threatened species and populations, better integration of genomic and ecological data, and stronger connections to conservation planning to preserve genetic connectivity and diversity in rapidly changing landscapes.

## Graphical abstract

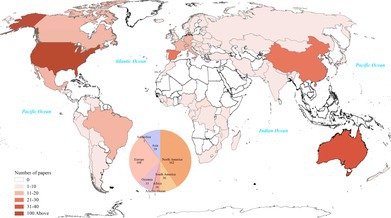

## 1 Introduction

Biodiversity serves as the fundamental basis for ecosystem functioning and human well‐being (Jenkins 2003; Maclaurin and Sterelny 2008), yet it is increasingly threatened by anthropogenic activities and climate change (Barnosky et al. 2011; Watson et al. 2019; Ceballos et al. 2020). The Living Planet Report 2025 indicates that global wildlife populations have declined by 70% over the past half‐century, primarily due to habitat degradation and loss (Tilman et al. 2001; Wei et al. 2014). These spatially heterogeneous environmental pressures have exceeded the capacity of traditional conservation approaches, necessitating landscape‐level strategies that account for ecological processes operating across multiple scales (Xue et al. 2011).

Landscape genetics, an interdisciplinary field integrating landscape ecology, spatial statistics, and population genetics, investigates how landscape composition and configuration influence the spatial distribution of genetic variation (Manel et al. 2003; Storfer et al. 2007). Since its formal introduction, the field has grown rapidly, with publication numbers increasing dramatically, reaching over fifty articles annually by 2008 (Storfer et al. 2007). A key distinction of this approach is the explicit quantification of landscape heterogeneity effects on gene flow and genetic variation, moving beyond simple isolation‐by‐distance tests (Holderegger and Wagner 2008). The field has evolved to address diverse questions, including identifying barriers to dispersal, quantifying the effects of landscape variables on connectivity, and assessing temporal scales of genetic responses (Holderegger et al. 2010; Xue et al. 2011; Manel and Holderegger 2013). Recent advances in genomic technologies have enabled landscape genomics approaches that integrate neutral and adaptive genetic variation, providing more precise insights into species‐specific responses to environmental heterogeneity.

Despite its growing importance in conservation biology, emerging evidence suggests persistent biases in research coverage (Bonnet et al. 2002; Clark and May 2002). The majority of landscape genetic studies have been conducted in North America and Europe (Storfer et al. 2007). Within taxonomic groups, tetrapods, including amphibians, reptiles, birds, and mammals, have received disproportionate attention compared to plants, fishes, and invertebrates (Donaldson et al. 2016; Kardong 2002; Waits et al. 2015). Several factors may contribute to this disparity: (1) terrestrial tetrapods often exhibit greater sensitivity to landscape fragmentation due to specific habitat requirements and thermophysiological constraints (Rivera‐Ortíz et al. 2015); and (2) tetrapods have historically received more funding and attention in conservation research compared to other taxonomic groups (Lindken et al. 2024; Moura et al. 2024).

Methodological challenges further constrain the field's progress: many studies lack explicit research design for evaluating the effect of landscape pattern on genetic structure, genetic structure is often analyzed regarding contemporary landscapes but may reflect historical landscapes, misleading results could be produced when limited loci are matched roughly to a combination of environmental variables, and many statistical methods could be inappropriate for landscape genetics (Storfer et al. 2007). Data gaps compound these issues, as continuing limited data standardization, ongoing flux in taxonomic nomenclature, and non‐random missingness in attribute values constrain integrative research and potentially cause biased inference (Moura et al. 2024). Furthermore, recent meta‐analyses demonstrate that habitat fragmentation affects genetic diversity differently across tetrapod groups: amphibians and mammals show strong negative effects on heterozygosity and allelic richness, while birds and reptiles exhibit more variable patterns of inbreeding response (Rivera‐Ortíz et al. 2015). These biases hinder the development of comprehensive conservation strategies applicable across diverse tetrapod lineages and ecosystems.

Here, we conducted a review of the scientific literature aiming to (1) identify the tetrapod groups that have been most studied; (2) evaluate the geographic coverage of existing research; (3) summarize the research questions and analytical methods commonly used; and (4) highlight key landscape factors and environmental variables associated with genetic variation. Additionally, we propose future research directions to enhance the field's theoretical and applied contributions.

## 2 Method

### 2.1 Literature Search and Review

We conducted a global systematic review of landscape genetics studies published in English between 2003 (when the term was first introduced) and December 2025. Our search encompassed three major scientific databases: Web of Science Core Collection (WOSCC), Scopus, and PubMed, using the search query TS = ((“landscape genetics” OR “landscape genomics” OR “functional connectivity” OR “isolation by resistance”) AND (“vertebrate” OR “mammal” OR “bird”, “amphibian” OR “reptile” OR “tetrapod”) NOT (“fish”)), where TS represents topic field (title, abstract, and keywords). This literature search method has been proven effective in different review works (Storfer et al. 2007; Dyer 2015; Flores‐Manzanero and Vázquez‐Domínguez 2019; Rico 2019), which also allows for systematic and reproducible analysis, although it certainly excludes some works. All literature retrieval and extraction were completed on January 10, 2026. Initial searches yielded 343, 280, and 105 records from WOSCC, Scopus, and PubMed, respectively. After merging results, duplicate records were removed. Records were then screened in two stages: (i) title and abstract screening, followed by (ii) full‐text assessment. We excluded studies that (1) were not original peer‐reviewed research articles (e.g., reviews, meta‐analyses, and methodological/theoretical papers without empirical data), (2) did not focus on vertebrate species or terrestrial/freshwater landscape contexts (e.g., marine or fish‐focused studies, non‐target taxa), or (3) did not address landscape genetics/genomics or related connectivity/resistance‐based analyses (e.g., studies mentioning keywords but focusing on unrelated genetic topics such as population genetics without spatial landscape inference). After these exclusions, we retained 385 primary research articles for detailed analysis.

### 2.2 Data Management and Analysis

Each selected study underwent thorough examination to verify it met our inclusion criteria: empirical research on tetrapods that explicitly examined relationships between landscape features and genetic variation. For these studies, we systematically recorded multiple characteristics including: publication date; sampling country; IUCN Red List conservation status; taxonomic status (species, genus, family, and order); and the molecular markers employed. Research focus areas were categorized into seven thematic types: landscape connectivity, population structure, genetic diversity, gene flow, comparative analyses, association studies between genetic and environmental variables, and investigations of local adaptation (Flores‐Manzanero and Vázquez‐Domínguez 2019). In addition, we summarized landscape factors that affect genetic variation, including habitat permeability, topography, artificial landscapes, temperature and precipitation. The topographic variables considered included elevation, slope, and physical barriers, reflecting isolation by distance. Habitat permeability encompassed soil wetness, canopy cover, land cover type, and proximity to streams (Cline and Hunter Jr 2014; Gîlea and Pătru‐Stupariu 2025). Artificial landscape factors incorporated timber harvest areas, agricultural lands, roads, residential zones, and buildings (Gonzalez‐Abraham et al. 2007). Temperature and precipitation effects were captured through measures of frost‐free period, heat load index, and growing season precipitation (Trumbo et al. 2013; Flores‐Manzanero and Vázquez‐Domínguez 2019). This comprehensive data collection framework enabled us to assess global patterns and trends in landscape genetics research of tetrapod, while accounting for taxonomic, methodological, and geographical bias across studies conducted over two decades (Table S1).

To quantify temporal variation in annual publication output across the 20‐year study period (2003–2025), a non‐parametric Kruskal‐Wallis one‐way analysis of variance was implemented. The response variable was the annual number of published landscape genetics papers, and the predictor variable was calendar year (categorical grouping variable). The test was specified with a default significance level of *α* = 0.05 to detect overall temporal heterogeneity in publication volume. A Wilcoxon rank‐sum test (Mann–Whitney *U* test) was used to compare publication output between two temporal phases: the early stage (2003–2014) and the late stage (2015–2025). The response variable was annual publication count, and the grouping predictor was study period. A negative binomial generalized linear model (GLM) was applied with package “mass” to evaluate temporal trends in publication counts for each research theme (Ripley et al. 2013). For each thematic category, the model was fitted with annual publication count as the discrete response variable, and continuous calendar year as the sole predictor variable. A negative binomial error distribution was specified to account for overdispersion in count data (residual variance greater than the mean), with a log link function for model fitting. Model significance was assessed via likelihood ratio tests. Chi‐squared goodness‐of‐fit tests were performed for two core analytical objectives: (1) to evaluate whether publication frequency was evenly distributed across the seven research themes (response variable: observed publication counts per theme; expected values: equal theoretical distribution across all themes); (2) to assess whether research effort distribution across IUCN conservation categories deviated from global species baseline distributions (response variable: observed study counts for each IUCN category; expected values: standardized species richness proportions per IUCN category derived from official IUCN tetrapod species datasets). All chi‐squared tests were run with *α* = 0.05. When significant differences were detected, pairwise Chi‐square comparisons with Bonferroni correction were conducted using the “RVAideMemoire” package (Hervé 2017). The observed number of studies in each category was compared against expected frequencies derived from the global distribution of IUCN‐assessed tetrapod species across conservation categories. Standardized residuals were examined to identify which categories were over‐ or under‐represented in the literature.

Heatmaps were used to visualize trends in the distribution of genetic research themes across tetrapod taxa. Literature counts were summarized for categories by orders across seven genetic research themes, including genetic connectivity, genetic diversity, population structure, gene flow, comparative analysis, association analysis, and local adaptation. Heatmaps representing the number of publications for taxa versus research themes were generated using the R packages ComplexHeatmap and RColorBrewer (Neuwirth 2014; Gu 2022). This visualization allowed for intuitive comparison of research intensity across both taxa and thematic categories. To determine whether research themes were evenly represented across taxa, chi‐square goodness‐of‐fit tests were conducted to compare observed publication counts against expected equal distributions. All statistical analyses and visualization procedures were performed in Rstudio interface of R v3.6.1 (Rstudio Inc., Massachusetts, USA).

For bibliometric keyword network analysis, VOSviewer v1.6.14 (Van Eck and Waltman 2010) was used to construct term co‐occurrence networks. The analysis dataset comprised all standardized keywords extracted from the titles and abstracts of the 385 included studies. The network parameter was set to full counting of term co‐occurrences, with a minimum term occurrence threshold of five to filter low‐frequency invalid terms. Cluster analysis was performed using the default VOSviewer clustering algorithm to classify core research topics and identify knowledge structure and emerging research hotspots.

## 3 Results

### 3.1 Overview of Tetrapod Landscape Genetics

**FIGURE 1:**
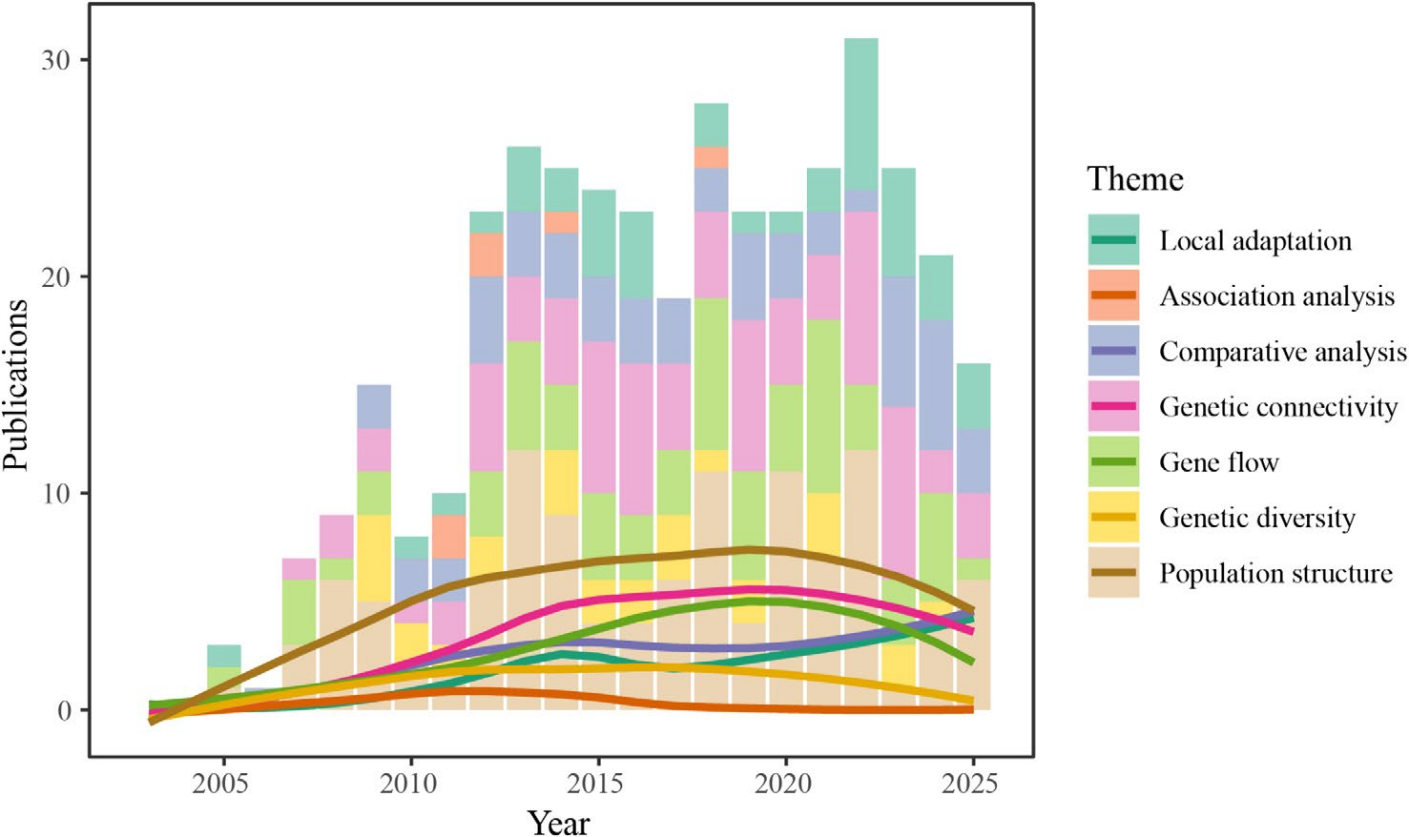
Annual publication counts across research themes in tetrapod landscape genetics, 2003–2025. Stacked bars represent the number of published studies per year, partitioned by research theme following our thematic classification: Local adaptation, association analysis, comparative analysis, genetic connectivity, gene flow, genetic diversity, and population structure. Smoothed lines correspond to the fitted temporal trends in publication volume for each individual theme, generated from negative binomial generalized linear models fitted to the annual count data.

A total of 385 papers were retrieved and analyzed to evaluate temporal trends in number of papers from 2003 to 2025 (Figure 1 and Table S2). There was a significant increasing trend over the study period according to the Kendall rank correlation analysis (tau *b* = 0.56, *p* < 0.01). The research themes with the highest number of publications per year varied over time: Structure analysis led in most years including 2008 (six papers), 2012 (six papers), 2013 (12 papers), 2014 (nine papers), 2018 (11 papers), 2020 (11 papers), and 2022 (12 papers); Genetic connectivity was dominant in 2022–2023 with eight papers each year; Genetic diversity peaked in 2009 with 4 papers; Gene flow reached its maximum in 2021 with eight papers. In contrast, association analysis consistently had the fewest publications, remaining at zero or one paper throughout the entire study period (only reaching two papers in 2011 and never exceeding this number). Moreover, a comparison between the earlier period (2003–2014) and the later period (2015–2025) revealed a significant increase in papers after 2014 (Wilcoxon rank‐sum test, *W* = 21.5, *p* < 0.01). The mean annual number of papers increased markedly across successive periods, being lowest from 2003 to 2008 (three papers per year), increasing during 2009–2014 (18 papers per year), reaching a higher level during 2015–2020 (23 papers per year), and remaining relatively stable but slightly higher during 2021–2025 (24 papers per year). Papers peaked in 2018 and 2022, with 28 and 31 papers respectively. No original papers on tetrapod landscape genetics were published in 2003, but papers increased steadily during the following decade, reaching 23 papers by 2012.

### 3.2 Taxonomy and Geographic Region

**FIGURE 2:**
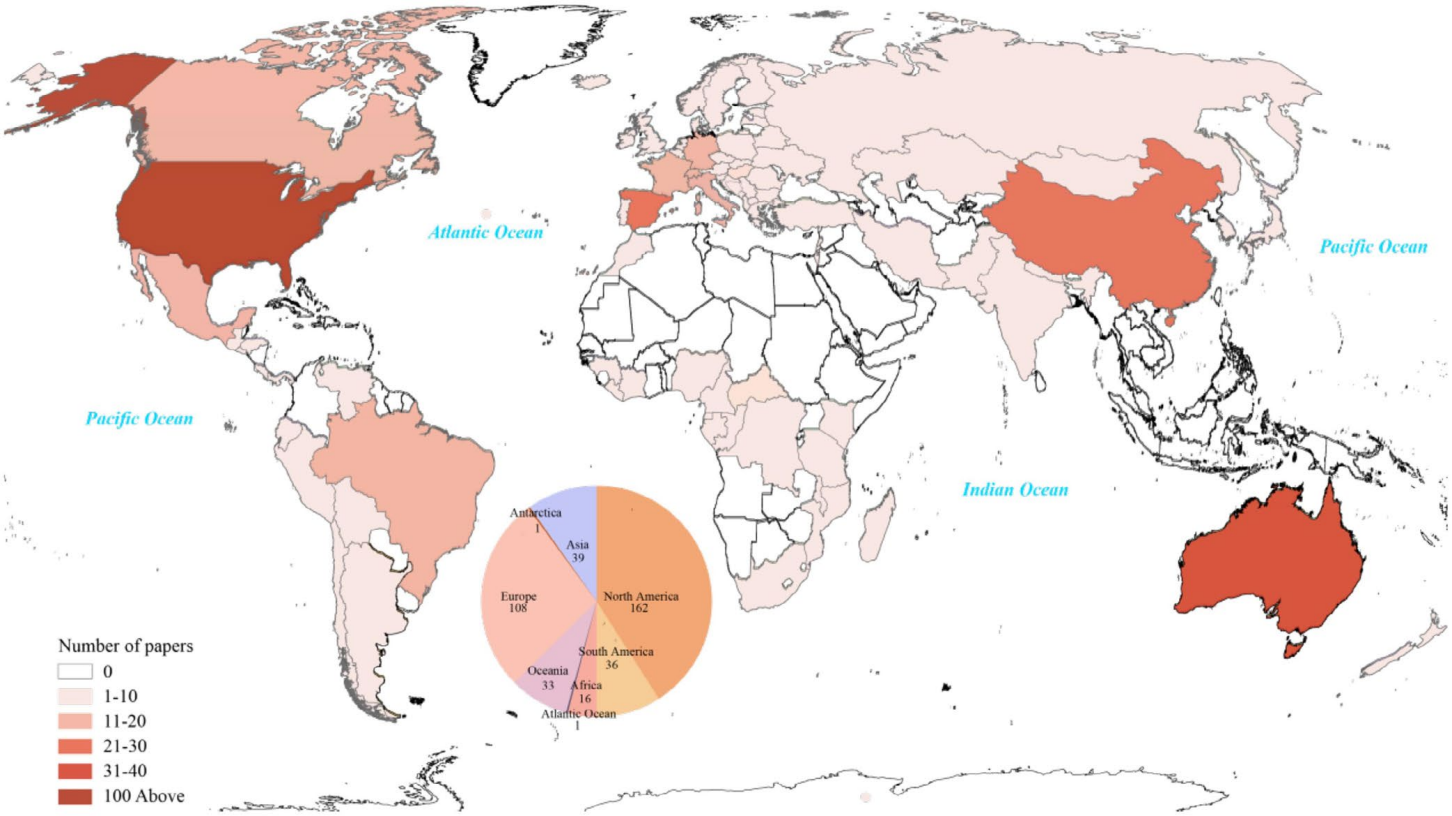
Geographic distribution of tetrapod landscape genetics between 2003 and 2025.

Across all included studies, 434 species were documented from 87 countries across eight major geographic regions, representing 294 genera, 138 families, and 34 orders (Figure 2). The spatial distribution of records was broad but uneven, with North America contributing the largest number (*n* = 162), followed by Europe (*n* = 108), Asia (*n* = 39), South America (*n* = 36), and Oceania (*n* = 33), while Africa was comparatively underrepresented (*n* = 16). Only single records were reported from the Atlantic Ocean (*n* = 1) and Antarctica (*n* = 1). In terms of taxonomic composition, amphibians were the most frequently reported group (*n* = 164), followed by mammals (*n* = 114), birds (*n* = 100), and reptiles (*n* = 29). Amphibians were particularly prevalent in North America (*n* = 89) and Europe (*n* = 42), whereas mammals were relatively well represented in North America (*n* = 33), Europe (*n* = 34), and Asia (*n* = 15). Birds showed a more balanced distribution among regions, with notable records from North America (*n* = 33), Europe (*n* = 26), South America (*n* = 13), and Oceania (*n* = 13). Reptiles represented the smallest proportion overall, occurring mainly in North America (*n* = 11), Europe (*n* = 8), and Oceania (*n* = 6). Moreover, the United States accounted for the highest number of records (*n* = 138), far exceeding other countries, including Australia (*n* = 31), China (*n* = 23), Spain (*n* = 21), Brazil (*n* = 19), Canada (*n* = 19), Mexico (*n* = 19), France (*n* = 18), Germany (*n* = 16), and Italy (*n* = 15), while many additional countries contributed smaller numbers of records.

The distribution of research effort across IUCN conservation categories differed significantly from expectations based on the global distribution of species (*χ* ^2^ = 56.31, df = 5, *p* < 0.001). Most species were classified as Least Concern (LC; *n* = 298) with a portion belonging to threatened or near‐threatened categories, including Vulnerable (VU; *n* = 45), Endangered (EN; *n* = 24), and Critically Endangered (CR; *n* = 10), while 37 species were categorized as Near Threatened (NT). Only a small number of records corresponded to species listed as Data Deficient (DD; *n* = 2), Not Evaluated (NE; *n* = 1), or Not Applicable (NA; *n* = 1). Standardized residuals indicated that LC and VU species were significantly over‐represented in the literature (LC: 3.60; VU: 2.50), whereas EN and CR species were under‐represented (EN: −2.03; CR: −2.32).

### 3.3 Theme Distribution

**FIGURE 3:**
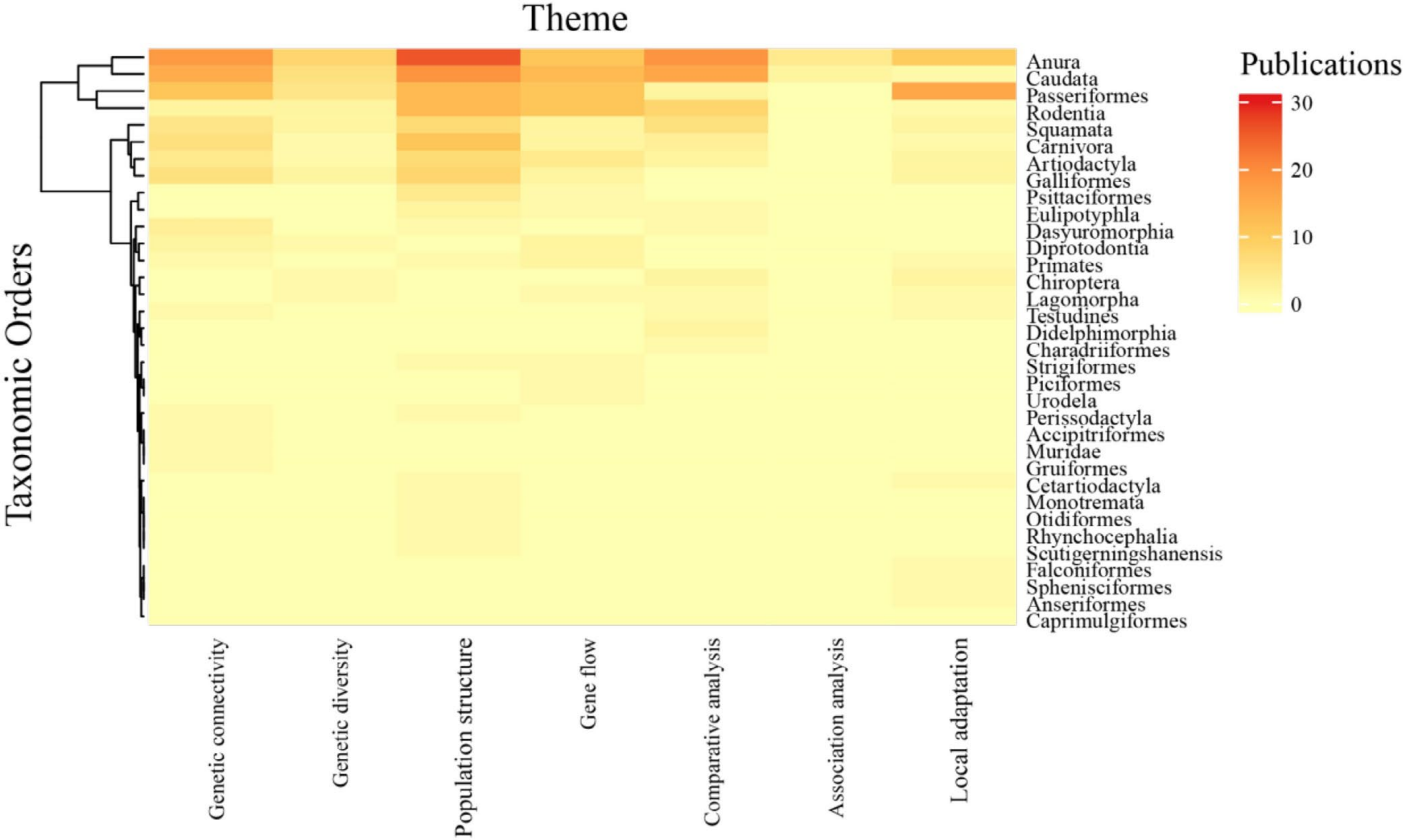
Clustered heatmap of literature count in research themes and categories by orders from literature. Rows represent tetrapod taxonomic orders, and columns correspond to the seven predefined research themes. Color intensity corresponds to the number of published studies falling into each taxonomic order–research theme combination. Hierarchical clustering was applied to group taxonomic orders with similar thematic research focus patterns.

Among all themes, population structure (*n* = 118) and genetic connectivity (*n* = 79) accounted for the highest proportions of studies, followed by comparative analysis (*n* = 65), gene flow (*n* = 63), and local adaptation (*n* = 43) (Figure 3 and Table S3). In contrast, genetic diversity (*n* = 28) and association analysis (*n* = 6) were markedly less represented across the literature. Across taxonomic groups, amphibians dominated the literature, with Anura (*n* = 96) and Caudata (*n* = 72) contributing the largest numbers of papers and showing broad thematic coverage across nearly all research categories (*χ* ^2^ = 1365.3, df = 33, *p* < 0.01) (Figure 3). Birds and mammals showed moderate representation, particularly Passeriformes (*n* = 58), which displayed a strong bias toward local adaptation studies (*n* = 16), and Rodentia (*n* = 37), where research was concentrated in population structure (*n* = 13) and gene flow (*n* = 11). Several carnivorous and ungulate groups, including Carnivora (*n* = 24) and Artiodactyla (*n* = 20), showed relatively balanced but overall lower research output. Reptile groups such as Squamata (*n* = 24) displayed moderate representation primarily in population structure and comparative analysis, while other taxa were represented by only one or two studies.

The total number of publications differed significantly among the seven research themes according to a Chi‐square goodness‐of‐fit test (*χ* ^2^ = 138.47, df = 6, *p* < 0.01) (Figure 1). Pairwise Chi‐square comparisons with Bonferroni correction further showed that the “population structure” domain contained significantly more publications than most other domains (*p* < 0.01), while “association analysis” had significantly fewer publications compared with all other themes (*p* < 0.01). Other domains such as “genetic connectivity” and “gene flow” showed intermediate publication counts and were not significantly different from some themes after Bonferroni correction. Temporal visualization indicates that research attention shifted over time, with “population structure” and “genetic connectivity” becoming dominant topics after 2012, while “local adaptation” showed a marked increase after 2020. In contrast, “association analysis” remained consistently rare across the entire study period.

### 3.4 Statistical‐Spatial Methods

In tetrapod landscape genetics studies, six molecular markers used separately, including simple sequence repeats (SSR, *n* = 231), single nucleotide polymorphisms (SNPs, *n* = 70), mitochondrial DNA (mtDNA, *n* = 30), amplified fragment length polymorphisms (AFLP, *n* = 5), nuclear DNA (nDNA, *n* = 1), and randomly amplified polymorphic DNA (RAPD, *n* = 1) (Table S4). In addition to single‐marker approaches, several studies combined multiple markers to improve analytical resolution. Common combinations included mtDNA with SSR (*n* = 29), mtDNA with nDNA (*n* = 8), and SNPs with mtDNA (*n* = 3). More complex combinations, such as mtDNA‐SSR‐nDNA (*n* = 4), SSR‐SNPs (*n* = 1), and mtDNA‐AFLP (*n* = 1), were occasionally reported. In contrast, AFLP (*n* = 5) and RAPD (*n* = 1) were rarely used due to their labor‐intensive procedures and dependence on radioactive markers (Grover and Sharma 2016).

In almost all studies, at least two analytical methods were used for assessing the genetics‐landscape relationship. The degree of genetic differentiation among populations across heterogeneous landscapes is commonly quantified using population structure analyses such as STRUCTURE (*n* = 170), assignment analysis (*n* = 71), principal component analysis (PCA) (*n* = 108), and analysis of molecular variance (AMOVA) (*n* = 102) (Zalewski et al. 2009; Wang et al. 2011; Fabbri et al. 2013). Genetic distance metrics (*n* = 157) are also widely used to evaluate differentiation among populations. To investigate the relationships between genetic variation and landscape or geographic features, a range of spatial statistical approaches are applied, including Mantel tests (*n* = 119), partial Mantel tests (*n* = 97), isolation by distance (IBD) analysis (*n* = 132), and spatial autocorrelation analysis (*n* = 134), which assess correlations between genetic and geographic distances (Sarasola‐Puente et al. 2011; Pereoglou et al. 2013). In addition, regression‐based frameworks such as generalized linear models (*n* = 98) and mixed‐effect models (*n* = 114) are frequently employed to quantify the influence of environmental and spatial variables on genetic patterns (Vernesi et al. 2016; Dutcher et al. 2020; Merchant et al. 2024). Landscape resistance and genetic connectivity are often modeled using resistance matrices (*n* = 100) and resistance distances (*n* = 107), together with connectivity approaches such as least‐cost path analysis (*n* = 97) and circuit theory models (*n* = 94), while simulation‐based methods (*n* = 91) are also applied to evaluate alternative landscape scenarios and their potential effects on gene flow (Spear and Storfer 2010a; Moore et al. 2011; Trumbo et al. 2013).

### 3.5 Landscape Factors That Determine Genetic Variation

The results on tetrapods indicated that habitat permeability exhibited the strongest association with genetic variation among the evaluated variables, with 208 papers highlighting its role, followed by topography (170 papers) and artificial landscape features (122 papers). Temperature and precipitation factors were less frequently associated (92 papers), while a small proportion of cases (16 papers) showed no clear association with any environmental factor.

### 3.6 Keywords Dynamics

**FIGURE 4:**
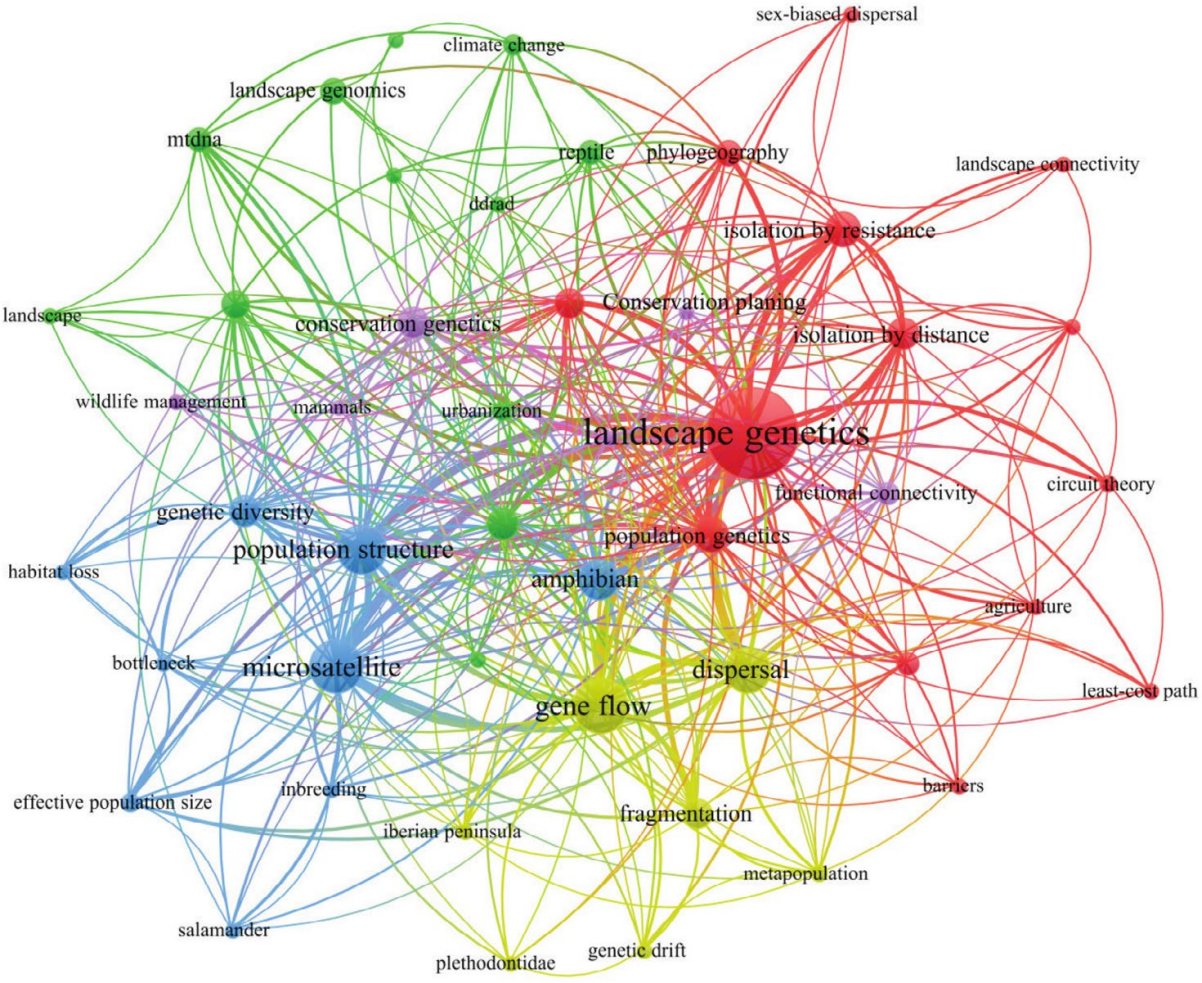
A co‐occurrence network of terms and their links extracted in VOSviewer. A binary counting approach (presence or absence of a term per publication) was employed with a minimum term occurrence count of five and a manually curated thesaurus file to filter irrelevant terms. Phrase occurrence was used for weighting the network. The clusters are separated into landscape connectivity and resistance (red), landscape genomics and environmental adaptation (green), population genetic structure and molecular marker analysis (blue), ecological and evolutionary processes (yellow), and conservation and management applications (purple).

The keyword co‐occurrence network reveals five main clusters that represent the major research directions in landscape genetics (Figure 4). The red cluster mainly focuses on how landscape features influence genetic connectivity among populations. Studies in this group often examine how barriers, habitat fragmentation, and landscape resistance affect organism movement and gene exchange, and they frequently apply spatial modeling approaches such as circuit theory or least‐cost path analysis to understand connectivity patterns. The green cluster reflects the integration of landscape genetics with evolutionary and genomic research, emphasizing topics such as adaptation, climate change, urbanization, and landscape genomics, often using modern genomic tools to explore how environmental change shapes genetic differentiation. The blue cluster is largely related to population genetic analysis using molecular markers, particularly studies on amphibians that investigate genetic diversity, population structure, inbreeding, and demographic events such as bottlenecks. The yellow cluster highlights key ecological and evolutionary processes, focusing on dispersal, gene flow, genetic drift, and metapopulation dynamics, which are important for understanding how species maintain genetic variation in fragmented landscapes. Finally, the purple cluster centers on conservation applications, linking landscape ecology with conservation genetics, functional connectivity, and wildlife management, demonstrating the application of landscape genetics methods in supporting conservation planning and biodiversity management.

## 4 Discussion

Since the term was first introduced, researchers have steadily developed the theories, methods, and analytical tools behind it (Manel et al. 2003; Holderegger et al. 2006; Balkenhol et al. 2016). Landscape genetics is now widely used in science to study how environmental factors influence the structure and genetic variation of natural populations (Manel et al. 2010; Dyer 2015). Our review demonstrates that, despite substantial growth in the field over the past two decades, research remains unevenly distributed across geographic regions, taxonomic groups, conservation priorities, and methodological approaches. These biases have important implications for both the development of landscape genetics and its application to biodiversity conservation.

### 4.1 Biases in Taxonomic, Geographic and Conservation Focus in Tetrapods Landscape Genetics

Tetrapods comprise 33,281 species, including 7238 amphibians (Jetz and Pyron 2018), 384 chelonians and crocodilians (Colston et al. 2020), 9755 squamates and tuatara (Tonini et al. 2016), 9993 birds (Jetz et al. 2012), and 5911 mammals (Upham et al. 2019). Of these, ~4000 are located in North America (Burgin et al. 2018). Our results showed that this region is the best represented in landscape genetics studies with tetrapods, supported by long‐term foundational case studies such as the landscape connectivity analysis of spotted tiger salamanders ( *Ambystoma tigrinum melanostictum* ) (Spear et al. 2005), one of the earliest empirical landscape genetic investigations for this region. In addition, we found scarce research efforts done on landscape genetics involving the tetrapods of Asia, South America, Africa and Oceania, which is consistent with previous results on mammalian landscape genetics in the American ecosystem (Flores‐Manzanero and Vázquez‐Domínguez 2019). Because tropical and developing regions harbor a disproportionate share of global biodiversity and contain many endemic species experiencing rapid habitat transformation (Kerr and Burkey 2002), the current geographic bias limits our understanding of how landscape processes influence genetic connectivity under a wider range of ecological conditions (Manel et al. 2003).

There is a disproportionate research focus on Anura and Passeriformes, two vertebrate groups that are widely used as model taxa in landscape genetics. Anurans are particularly well suited to landscape genetic studies because of their limited dispersal ability, strong habitat fidelity, permeable skin, and high sensitivity to fragmentation between aquatic and terrestrial habitats (Pitt et al. 2017; Reyne et al. 2023). In contrast, passerine birds show substantial variation among species in dispersal capacity, habitat preferences, and tolerance of human disturbance, making them valuable for comparing how different landscape features influence gene flow (Pavlacky Jr et al. 2009; Amos et al. 2014). While research on these two groups has provided important insights into the mechanisms underlying landscape‐driven genetic differentiation, their dominance in the literature limits our broader understanding of how different life‐history traits, such as long‐distance migration, fossorial lifestyles, or low dispersal ability in reptiles, shape landscape genetic patterns across the full diversity of tetrapods.

An equally important finding is the mismatch between research effort and conservation need. LC received disproportionately more research attention than threatened species, mirroring patterns observed in other taxa globally (Jarić et al. 2015; Conenna et al. 2017; Lambert et al. 2023). The overrepresentation of LC and VU species in the literature likely reflects their wider distributions and relative accessibility, rather than the priority of biological conservation. For instance, the EN and CR species in our dataset inhabit isolated or specialized habitats, often in remote or difficult‐to‐access areas, which constrains survey efforts and publication output (Reding et al. 2010; Magro et al. 2018; Sunny et al. 2022). Prioritizing research on EN and CR species is therefore critical to prevent further biodiversity loss (Arponen 2012).

### 4.2 Development of Methods in Tetrapods Landscape Genetics

In landscape genetics, the choice of molecular markers profoundly shapes our understanding of how species respond to environmental heterogeneity. While SSRs extensively utilized in landscape genetic studies historically, largely because genome‐wide data were not feasible at that time rather than because they represent superior markers, the rise of high‐throughput sequencing has made SNPs increasingly accessible, allowing researchers to examine thousands of loci across the genome. Recent empirical comparisons have demonstrated that SSR markers exhibit strong biases when evaluated against whole‐genome data, including ascertainment bias, uneven mutation rates across loci, and reproducibility challenges that can affect population parameter estimation (Pérez‐González et al. 2023; Skey et al. 2023). The shift toward genomic approaches enables detection not only of neutral population structure but also of variants under selection, providing insights into adaptive potential (Storfer et al. 2007; Balkenhol et al. 2017). For example, studies on urban Norway rats ( *Rattus norvegicus* ) have revealed fine‐scale genetic structuring linked to human‐made barriers, which SSR alone could not detect, highlighting the added resolution of SNP datasets (Combs et al. 2018). Similarly, research in desert bighorn sheep demonstrates that loci associated with adaptive traits move preferentially through continuous vegetation patches, illustrating how landscape features mediate gene flow for ecologically important genes (Creech et al. 2014).

Building on these advances in molecular markers, landscape genetics research has developed various analytical methods to explain how environmental heterogeneity shapes gene flow and population structure, ranging from classical population genetic methods like STRUCTURE and AMOVA to spatial analyses such as Mantel tests, isolation by distance, and spatial autocorrelation. For example, in fragmented forest landscapes, resistance distances derived from landcover features or human‐made barriers better explain genetic differentiation than simple Euclidean distances (Vignieri 2005). Regression‐based models, such as mixed‐effect models, can then quantify the influence of environmental gradients like elevation or soil type on genetic variation, revealing which landscape elements facilitate or hinder connectivity (Keighley et al. 2020; Daniel et al. 2023). Notably, integrating resistance surfaces with least‐cost path or circuit theory models allows the identification of corridors critical for maintaining gene flow (Storfer et al. 2007; Mcrae et al. 2008), as shown in studies of small mammals navigating urban matrices or amphibians moving through patchy wetlands (Prunier et al. 2013; Borja‐Martínez and Vázquez‐Domínguez 2024). These approaches not only clarify current patterns of population structure but also enable predictions about how future landscape changes, such as urban expansion or climate‐driven habitat shifts, might alter connectivity, providing actionable insights for conservation planning (Costanza and Terando 2019; Zeller et al. 2020).

### 4.3 Factors That Determine Genetic Variation in Tetrapods

Reviews across tetrapods reveal habitat permeability as the most significant predictor of genetic variation, particularly well‐represented in frogs (Measey et al. 2007; Spear and Storfer 2010b), followed by topographic variables and artificial landscape features. Variables related to habitat permeability, such as soil moisture, canopy cover, land cover type, and proximity to streams, were most frequently associated with genetic variation across studies (Decocq et al. 2021). These factors likely affect movement and dispersal by modifying the resistance or connectivity of habitats, thereby influencing gene flow among populations. Topographic variables, including elevation, slope, and physical barriers, were also widely identified as determinants of population structure, reflecting patterns consistent with isolation by distance or terrain‐mediated dispersal constraints (Hulva et al. 2017; Fernández De Larrea et al. 2021). In contrast, climatic variables such as temperature and precipitation were less frequently linked to genetic variation, but their influence was most evident in amphibians and some cold‐adapted or high‐latitude vertebrates, where physiological sensitivity to moisture and thermal conditions strongly constrains dispersal and habitat suitability. For instance, several amphibians such as the Western leopard toad ( *Sclerophrys pantherina* ), Coastal tailed frog ( *Ascaphus truei* ), and Yosemite toad (*Anaxyrus canorus*) showed genetic variation associated with precipitation or temperature‐related variables such as frost‐free periods (Measey and Tolley 2009; Spear et al. 2012; Maier et al. 2022). Similar climatic influences were also reported in cold‐environment or wide‐ranging species, including the Brown bear ( *Ursus arctos* ), Alpine shrew ( *Sorex alpinus* ), and Gray long‐eared bat ( *Plecotus austriacus* ) (Keis et al. 2012; Starcová et al. 2016; Razgour et al. 2018).

Additionally, artificial landscape features were commonly associated with population structure, elements such as timber harvest areas, agricultural lands, roads, residential zones, and buildings can either impede or redirect movement pathways, thereby generating genetic differentiation. For instance, the wall along the Mexico and the United States border is a significant barrier to dispersal and gene flow across populations (Flesch et al. 2010). Notably, physiological and ecological constraints can also shape population structure. For example, in western slimy salamanders ( *Plethodon albagula* ), resistance to gene flow is best explained by the rate of water loss, a key physiological limitation. Individuals exhibit reduced movement under extreme moisture conditions, ultimately driving genetic differentiation across the landscape (Peterman et al. 2014). Therefore, for biodiversity management, priority should be given to natural habitat corridors to maintain permeability, while human infrastructure requires targeted mitigation measures to offset diffusion barriers. The physiological thresholds of specific classifications further enable customized conservation planning, enabling practitioners to design humidity, or temperature‐friendly shelters for climate‐sensitive amphibians and cold‐adapted vertebrates in ongoing landscape transformations.

### 4.4 Prospects for Landscape Genetics Studies

Despite rapid advances in the past two decades, landscape genetics of tetrapods still faces major gaps in geographic coverage, taxonomic representation, and methodological integration. To fully realize the potential of this field, we propose several key directions for future research:

1. Most landscape genetics studies on tetrapods have been conducted in North America, followed by Europe, while Asia, South America, Africa, and Oceania remain poorly studied. Increasing research efforts in these regions would help provide a more balanced global understanding of how landscapes shape genetic variation across different ecological contexts.
2. Current studies are strongly biased toward certain model groups, particularly frogs, toads, and perching birds. Future work should broaden the taxonomic scope to include a wider range of tetrapod taxa and direct more research toward threatened species and populations to better capture the diversity of life histories and dispersal strategies influencing landscape genetic patterns.
3. The widespread availability of high‐throughput sequencing has rendered SNPs a dominant molecular tool superior to traditional markers. Genome‐wide SNP panels enable robust characterization of neutral population genetic structure while efficiently capturing signatures of landscape‐driven adaptive variation, delivering unprecedented resolution to disentangle how environmental landscapes shape evolutionary trajectories of wild populations.
4. Genetic patterns are often shaped not only by landscape structure but also by species ecology, including dispersal ability, habitat specialization, and demographic history. Future studies should integrate ecological and behavioral data to better explain why different species respond differently to similar landscape features.
5. Finally, future work should increasingly translate landscape genetic findings into practical conservation actions. Identifying dispersal corridors, landscape barriers, and areas critical for maintaining connectivity can support management decisions aimed at preserving genetic diversity and long‐term population viability.

## 5 Conclusion

Landscape genetics has become an important approach for understanding how environmental factors shape gene flow and population connectivity in tetrapods. Research over the past two decades shows that landscape features such as habitat permeability, topography, and human‐made barriers strongly influence genetic connectivity among populations. However, current studies are unevenly distributed geographically and taxonomically, with most research focused on North America and on frogs, toads, and perching birds. Future studies should expand to understudied regions and taxa, with particular attention to threatened species and populations, while integrating genomic tools, improved environmental data, and ecological information. Strengthening the connection between landscape genetics and conservation planning will also be essential, as this field can help identify key corridors, barriers, and habitats necessary to maintain genetic diversity and population persistence in rapidly changing environments.

## Author Contributions

**Tongtong Gu:** conceptualization (lead), funding acquisition (lead), software (equal), writing – original draft (lead), writing – review and editing (lead). **Xing Jia:** conceptualization (supporting), methodology (supporting), software (supporting), writing – original draft (supporting). **Yuxin He:** conceptualization (supporting), methodology (supporting), software (supporting), writing – original draft (supporting). **Zixin Lin:** conceptualization (supporting), methodology (supporting), software (supporting), writing – original draft (supporting).

## Funding

This research was funded by the National Natural Science Foundation of China (Grant 32500436), Yunnan Fundamental Research Projects (Grant 202601CI070016), and College Students’ Innovative Entrepreneurial Training Plan Program (Grant DC2025147).

## Conflicts of Interest

The authors declare no conflicts of interest.

## Supporting information


**Table S1:** The research database of all publications of tetrapod landscape genetics, after curation and inclusion of relevant information for further analyses.

**Table S2:** Annual counts of studies across seven research themes (genetic connectivity, genetic diversity, structure analysis, gene flow, comparative analysis, association analysis, and local adaptation) from 2003 to 2025. Values represent the number of papers for each theme in a given year. Zeros indicate that no relevant studies were recorded for that category in that year.

**Table S3:** The number of papers by order and research theme.

**Table S4:** The studies using different genetic markers.

## Data Availability

No datasets were generated or analyzed during the current study.
